# A Prolonged Nightly Fasting Plus Telehealth Coaching Intervention (PNF+) for Men on Androgen Deprivation Therapy for PCa: A Pilot Feasibility Randomized Controlled Trial

**DOI:** 10.3390/nu18071166

**Published:** 2026-04-07

**Authors:** Kuang-Yi Wen, Julianne Freedman, Kevin Kayvan Zarrabi, Rachel Slamon, Rita Smith, Jessica Liang, Patrick Mille, William J. Tester, William Kelly

**Affiliations:** Department of Medical Oncology, Thomas Jefferson University, Philadelphia, PA 19143, USA; julianne.freedman@jefferson.edu (J.F.);

**Keywords:** prolonged nightly fasting, cancer care, m-Health, dietary monitoring, dietary patterns

## Abstract

**Background/Objectives**: This study aimed to assess the feasibility and acceptability of a 3-month health coaching intervention to promote PNF and healthy diet for men on ADT for PCa. **Methods**: The study was carried out via a two-armed randomized controlled trial including 40 patients with PCa at a medical center in Philadelphia. During the 3-month period, the intervention group (PNF+) received health coaching utilizing an interactive text message system, and the control group received healthy eating text messages for the same duration. The outcome variables were feasibility and acceptability. **Results**: The PNF+ group (*n* = 27) had high adherence to health coaching (82%), picture response (85%) and moderate adherence to the PNF window (69%). The intervention was rated highly acceptable with no reported A/E associated with the intervention, and most participants planning to continue in some capacity. At 3 months, the PNF+ group had numerically lower BMI (29.1) and body weight (195.2 lbs) compared to the control group (*n* = 13; BMI 31.6, weight 223.3 lbs). Improvements in patient-reported outcomes were observed in both groups. FACIT-F scores (higher scores indicate less fatigue) increased in the PNF+ group (43.6 to 45.2) and in the control group (42.5 to 45.5). FACT-P scores (higher scores indicate better quality of life) increased in the PNF+ group (121.3 to 125.5) but decreased slightly in the control group (121.1 to 119.8). Between-group comparisons of change from baseline showed no statistically significant differences across outcomes (all *p* > 0.05). **Conclusions**: The intervention demonstrated partial feasibility and high acceptability. It was associated with numerically lower BMI and body weight and favorable changes in patient-reported outcomes, particularly quality of life; however, no statistically significant differences were observed between groups. These findings should be interpreted cautiously given the small sample size and require confirmation in larger, adequately powered trials.

## 1. Introduction

Prostate cancer (PCa) is the most common hormone-dependent, nondermatological malignancy in older men and the second leading cause of cancer mortality in the United States [1]. Androgen deprivation therapy (ADT) is the mainstay of treatment for advanced PCa, with a treatment trajectory potentially spanning over several years in a significant proportion of men [2]. While ADT is associated with survival advantages and a reduction in disease-specific mortality, the side effects of long-term treatment can greatly impact quality of life (QOL). Adverse effects include fatigue, reduced bone mineral density, weight gain, development of metabolic syndrome, insulin resistance, increased risk for adverse cardiovascular events, and diabetes [2]. It has been found that gaining more than 5% of one’s body weight post PCa diagnosis is associated with a 27% higher risk of all-cause mortality and a 65% increased risk of prostate cancer specific mortality (PCSM) [3]. Interventions targeting the effect of nutrition and lifestyle could play an important role in the reduction in treatment associated side-effects, delay of disease progression and improve QOL. Diet is one of the most important modifiable factors linked to cancer incidence and mortality, with 6% of global cancer deaths and 5.5% of cancer-related disability-adjusted life years (DALYS) attributable to dietary risk factors [4]. While much of the existing literature has emphasized what constitutes a healthy or unhealthy diet, studies have begun to explore the significance of fasting-based interventions in cancer care.

Considerable evidence demonstrates that fasting and alterations in meal timing can have an impact on multiple health outcomes. Prolonged nightly fasting (PNF) is a form of intermittent fasting that involves fasting for an extended period of time, with 8 h of eating and 16 h of overnight fasting (16:8) as a common approach [5]. The mechanism linking PNF and metabolic regulation can be traced to the circadian clock, controlling the expression of several genes involved in glucose metabolism and cellular homeostasis [5]. Fasting has been shown to reduce circulating insulin and insulin-like growth factor-1 (IGF-1), which in turn inhibits the PI3K/Akt/mTOR signaling axis and activates autophagy. These processes are associated with enhanced DNA repair and tumor suppression [6,7,8]. Studies in rodent models show that PNF patterns have preventative and therapeutic effects on metabolic diseases and protection against fat accumulation and adipose inflammation [9]. Several small-scale trials of various intermittent fasting and PNF interventions in cancer patients and survivors have demonstrated that a prolonged duration of nighttime fasting, such as a 16:8, is feasible and safe, with improvements in weight management, chemotherapy toxicity and a reduction in cardiovascular disease (CVD) risk and fatigue [10,11,12,13].

Further, digital interventions targeting diet can be a promising tool to track daily dietary behaviors and are advantageous over traditional methods of diet tracking, such as food diaries or questionnaires [14]. Utilizing technology can reduce costs and burden associated with on-site intervention participation, as well as scaling up more readily and cost effectively for maximum reach [15]. Additionally, health coaching is a patient-centered approach for disease management, focusing on patients’ behaviors and decisions [16]. Dietary interventions may require higher levels of motivation from participants, and health coaching may act as a tool to maintain motivation and promote patient self-efficacy [17].

As one of the first, the objective of this randomized controlled trial (RCT) is to test the feasibility and acceptability of a telehealth coaching and interactive text-integrated intervention to promote a 16 h prolonged nightly fast (PNF+) among men receiving ADT for PCa.

## 2. Materials and Methods

The PNF+ study was a pilot RCT assessing the feasibility and acceptability of a 3-month telehealth coaching and interactive text-integration promoting a 16:8 PNF (PNF+) versus 3 months of receiving messages promoting healthy eating among men on ADT for PCa (Healthy Diet AC).

### 2.1. Recruitment Strategies

Study staff screened patient medical records based on diagnosis and treatment regimen. Clinic schedules, electronic medical records (EMR) and prescription databases were used to identify eligible participants. The study team developed flyers and brochures to be displayed in patient lobby areas and distributed through community settings, organizations, and stakeholders. The team utilized Jefferson Enterprise Business Intelligence and EPIC teams for data extraction requests. Eligible participants were reached through phone, email, text message, or in person at clinics. Participants were consented through REDCap eConsent or paper-based consent.

### 2.2. Randomization Procedures

After consent and the completion of baseline assessments, participants were randomized in a 2:1 ratio to PNF+ or Healthy Diet AC group using a computer-generated allocation sequence prepared prior to enrollment. A 2:1 randomization ratio was selected to allow greater exposure to the intervention, which is appropriate for pilot feasibility studies aimed at evaluating adherence, engagement, and implementation processes. Assignments were concealed until eligibility was confirmed and baseline measures were completed.

### 2.3. Inclusion and Exclusion Criteria

Patients must meet all of the following in order to be eligible to participate in the study: ≥18 years of age, provide a signed and dated informed consent form, had a histological diagnosis of PCa (Stage IIA-IVB), and be beginning or currently receiving ADT, with an anticipated duration of ≥3 months (no restriction on prior duration of therapy) (including medical castration with LHRH-agonist-antagonist androgen receptor modulators). The ADT is defined as: (a) Gonadotropin Releasing Hormone (GNRH) agonist (including leuprolide [Lupron/Eligard], goserelin [Zoladex], triptorelin [Trelstar], histrelin [Vantas], and abiratirone [Zytiga]) alone; (b) GNRH agonist with oral androgen receptor blockade (including bicalutamide [Casodex], flutamide [Eulexin], and enzalutamide [Xtandi]), and (c) GNRH agonist, oral androgen receptor blockade, and 5-alpha reductase inhibitors. Patients on androgen receptor modulators and second-generation androgen receptor antagonists qualified. Intermittent ADT is allowed if the overall duration is more than 3 months, and radiation therapy is allowed concurrently. Additionally, because ADT can contribute to an increase in body weight from treatment initiation [3] patients qualify with a body mass index (BMI) ≥ 25 kg/m^2^. Lastly, patients are willing and able to comply to the protocol for the study duration, able to speak, read and write English and have a mobile phone with text capability. Exclusion criteria included inability to tolerate a normal diet, such as because of an active malabsorption syndrome like Crohn’s disease or major bowl resection, concurrent chemotherapy, ECOG score > 2, taking medications such as finasteride/dutasteride/saw palmetto and taking glyburide medication or insulin injection. To prevent risk of exacerbation or relapse, patients with eating disorders were excluded from this study.

### 2.4. PNF+ Group

Participants randomized to PNF+ participated in one-on-one coaching calls for 3 months, with 2 calls per week in months 1 and 1 per week in months 2 and 3. Participants were encouraged to fast for at least 12 h nightly, gradually increasing to 16 h by the third week. Participants interacted with a text platform to send photos of their meals and report when they begin and end their overnight fast and sleep behaviors. In the first session, the health coach would review the purpose of the study and ask specific questions to gain an understanding of baseline mealtimes, sleep and wake times, food preferences, emotional support strategies, food shopping habits, and any cultural/religious influence on eating. The coach used this information as a guide for setting up feasible eating and fasting times. In the follow-up sessions, the health coach would check progress through the texting platform, assess challenges in the fasting schedule, and adjust the schedule accordingly.

Following the fundamentals of the social cognitive theory, we utilized a stepwise strategy, focusing on building self-efficacy through establishing goals and reviewing performance to achieve an increased overnight fasting duration. During the first month, we followed aspects of cognitive behavioral therapy such as (1) goal setting and self-regulatory skill development, (2) clinical relevance knowledge re-emphasis, (3) self-monitoring and tailored feedback, (4) barriers identification and coping facilitator development, (5) positive reinforcement and (6) dietary suggestion in context. In the second month, we followed the latter principles with the addition of new behavior adaptation and maintenance. If patients were successful with their fasting goal, the coach would increase their fasting duration by 30 min to 1 h after discussion with the participants in the health coaching session.

**Figure 1:**
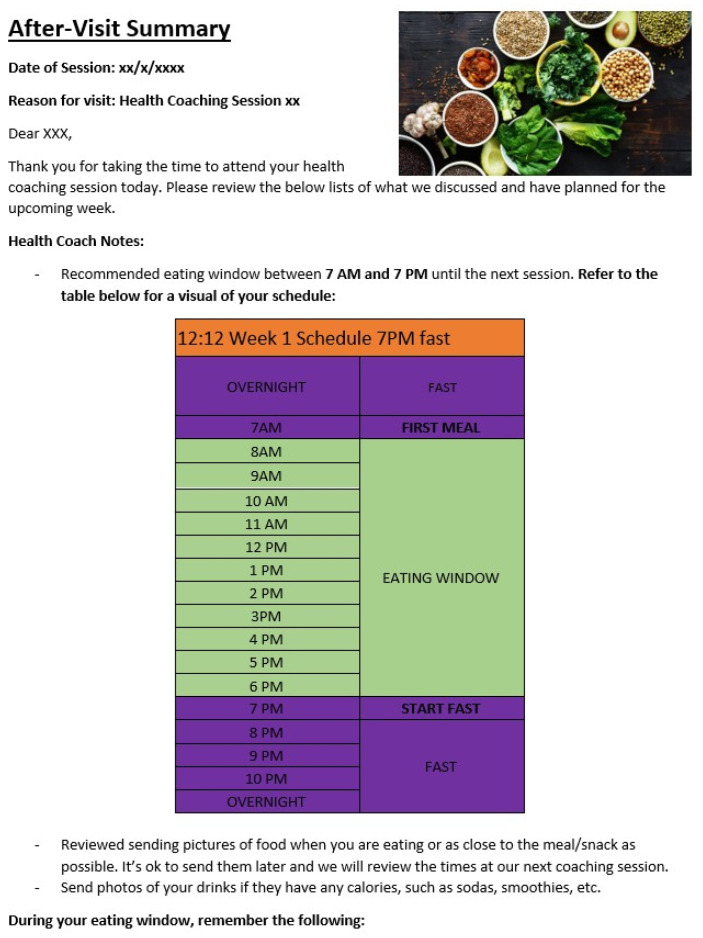
Health coaching after-visit summary with timetable sample.

For the fasting windows, water and medications were allowed at any time; however, coffee, tea, chewing gum and diet beverages were discouraged during the fasting window. The coach would also provide healthy eating suggestions from the American Institute for Cancer Research healthy eating website (e.g., AICR’s Foods that Fight Cancer Fact Sheet) based off participant behaviors. After each session, the health coach would send an after-visit summary to the participant to summarize the session and provide a visual of the fasting duration with a timetable (Figure 1). Two health coaches were assigned individual participant loads, with participants consistently seeing the same health coach throughout the study duration. One coach holds a master’s degree in public health with a focus area in food and nutrition, insecurity and dietary behaviors in population health. The second coach holds a master’s degree in health communication with several years of experience in the creation of health coaching content and direct delivery intervention in population science.

### 2.5. Healthy Diet (AC) Group

Participants randomized to the Healthy Diet AC group received educational information via text platform derived and extracted from American Institute for Cancer Research healthy eating website (e.g., AICR’s Foods that Fight Cancer Fact Sheet). Participants received text messages 3 times a day for 90 days to match the frequency of the intervention group.

### 2.6. Text Platform

**Figure 2:**
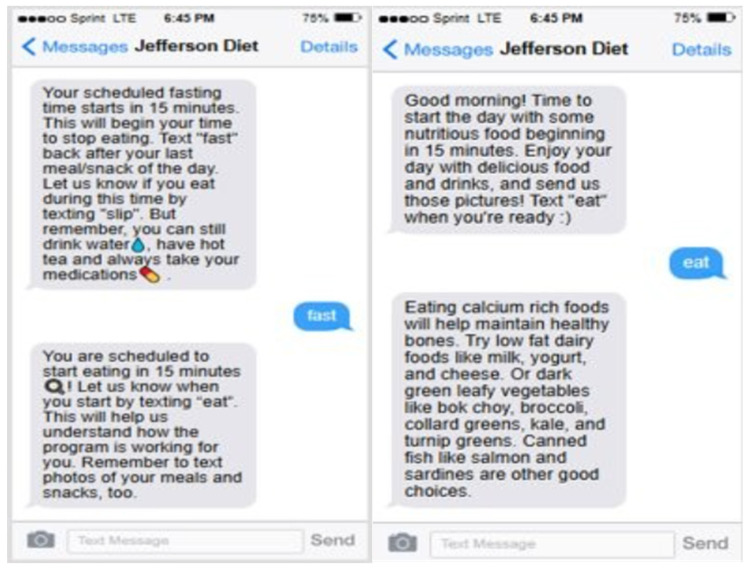
Text messaging interaction samples.

After the 1st coaching session, the health coach enrolled participants in the text platform using their mobile number, and they were instructed to begin the process the following day. The texting platform was scheduled to send an automated 15 min reminder text of the approaching eating and fasting window. The health coach would schedule the reminders to align with the set eating and fasting times. To monitor compliance in real-time, participants were required to text the words “EAT” and “FAST” to report when they begin and end their overnight fast (Figure 2). Along with the keywords, participants were also instructed to take pictures of all food and drinks of caloric value. Feedback on pictures and fasting/eating compliance was not given until the next scheduled health coaching session. Participants in the Healthy Diet AC group were enrolled in the texting platform following randomization.

### 2.7. Outcome Measures

#### 2.7.1. Feasibility

We assessed feasibility by calculating the accrual rate, attrition rate, compliance with PNF and health coaching attendance. The intervention is considered feasible with 70% of eligible patients we reach consenting and enrolling, 70% of enrolled participants completing post intervention follow-up and 70% of participants being compliant with 70% of the intervention days with suggested PNF (70% of 90 days = 63 days). These feasibility thresholds are consistent with commonly used benchmarks in pilot behavioral intervention studies. We assess the adherence to health coaching, PNF window and picture response on a scale of low to moderate to high.

#### 2.7.2. Acceptability

To gain further insight into feasibility and client perspective, the Client Satisfaction Questionnaire-8 item version (CSQ-8) was administered to both groups post-study [18]. The scores are summed across items with 4-items reverse scored, and the total scores range from 8 to 32 with a higher number indicating greater satisfaction [18]. No standardized cut-off values exist for CSQ-8; therefore, scores were interpreted descriptively, with higher scores indicating greater satisfaction. Qualitatively, a structured interview was conducted to assess the behavioral determinants, usefulness, and practicality of the intervention. Sample questions asked participants to identify the greatest benefit from the program, indication of the greatest challenge with compliance, and the driving force that encouraged them to participate in the program.

Acceptability was assessed with a weekly adverse events (A/E) checklist. The checklist was administered by the health coach to screen for potential issues that could be intervention related. Participants were instructed to contact the research staff if they experienced any new or worsening symptoms. This data was immediately reviewed by the research team, and A/E were reported as part of the study finding and to the institutional review board.

#### 2.7.3. Exploratory Outcomes

Fatigue and QOL were exploratory outcomes that were assessed at baseline, 1 month, 2 months and 3 months. Fatigue was measured by the Functional Assessment of Chronic Illness Therapy-Fatigue (FACIT-F) in short form [19]. The FACIT-F includes 13 items, such as “I feel fatigued” and “I feel weak all over.” Items are scored on a range from 0 to 52 with higher scores indicating a better QOL or less fatigue [19]. The Functional Assessment of Cancer Therapy-Prostate Cancer (FACT-P) (Version 4) was administered to assess the quality of life in patients with PCa [20]. This tool is stratified into 5 subscales, physical well-being, social/family well-being, emotional well-being, and additional concerns with a total score range from 0 to 156 with a higher score indicating a better quality of life [20].

### 2.8. Sample Size Consideration and Statistical Analysis

As a pilot feasibility study, a formal sample size calculation for hypothesis testing was not performed. The sample size was selected to evaluate feasibility metrics and generate preliminary estimates to inform future adequately powered trials. Analyses focused on estimation of feasibility parameters and preliminary effect sizes rather than formal hypothesis testing. Descriptive statistics (means, standard deviations, and frequencies) were used to summarize baseline demographic and clinical characteristics of participants. Descriptive statistics were also calculated for baseline and post-intervention values of body weight, BMI, FACIT-F, and FACT-P. Adherence to the intervention protocol was summarized using percentages to describe completion rates and compliance with the prescribed fasting window. For exploratory outcomes (body weight, BMI, FACIT-F, and FACT-P), analyses were conducted using change scores calculated as post-intervention minus baseline values for each participant. Between-group differences in change from baseline were assessed using independent t-tests, with results presented as mean differences, 95% confidence intervals, and effect sizes (Cohen’s d). *p*-values are reported descriptively to aid interpretation, and all findings should be considered exploratory.

## 3. Results

### 3.1. Recruitment and Retention

**Figure 3:**
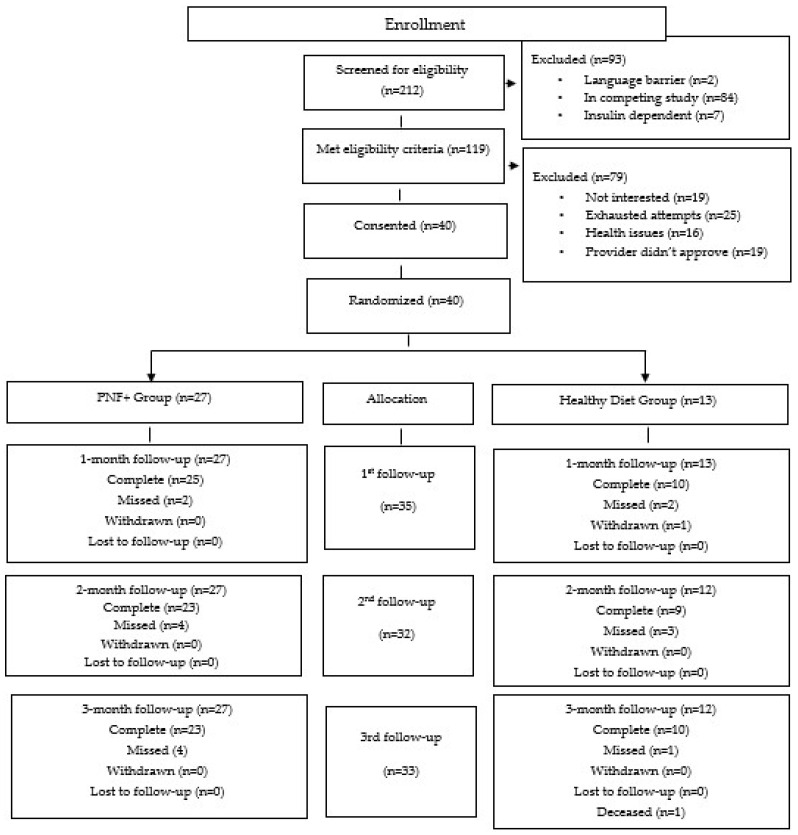
CONSORT diagram showing the flow of participants through each stage of the study, screening, eligibility assessment, enrollment, allocation and follow up.

Between 2 May 2024, and 11 March 2025, of the 212 patients screened, 119 met eligibility criteria. Of the 119 eligible patients, 40 patients were consented and randomized. Reasons for exclusion post-eligibility included no interest; all contact attempts exhausted, health issues or provider did not approve. Participants were randomly assigned to PNF+ (*n* = 27) or Healthy Diet AC (*n* = 13). The Healthy Diet AC group had one participant withdraw at the 1-month follow-up due to not wanting to send pictures or document food and one participant was deceased at the 3-month follow-up point (Figure 3).

### 3.2. Demographics

Table 1 presents the demographic and clinical characteristics of study participants. Among the 40 enrolled participants, the mean age was 69.5 ± 7.6 years, with no significant differences between the intervention and control groups (*p* = 0.68). Most participants identified as White in both groups (63.0% vs. 61.5%), with a similar proportion identifying as Black or African American (33.3% vs. 30.8%). Most participants reported an education below college (52.5%), followed by college or post-college education (47.5%), with no differences observed between groups (*p* = 1.00). Household income distributions differed between groups (*p* = 0.02). A greater proportion of participants in the intervention group reported annual incomes of $75,001+ (44.4% vs. 23.1%), whereas a higher proportion of control participants reported incomes in the $60,001–$75,000 range (30.8% vs. 0.0%). Given the small sample size, this difference should be interpreted cautiously as a potential imbalance at baseline rather than a definitive group difference. Medication was categorized as an LHRH agonist, androgen receptor inhibitor, androgen synthesis inhibitor, and bone-modifying agent. These categories were not mutually exclusive, and participants could receive more than one therapy. LHRH agonists were the most reported medications in both groups (85.2% in PNF+ vs. 76.9% in the control group). The use of androgen receptor inhibitors (29.6% vs. 23.1%) and androgen synthesis inhibitors (11.1% vs. 23.1%) varied between groups. The mean duration of medication use was 403.9 ± 590.0 days overall, with no statistically significant difference between groups (369.8 ± 604.8 days in PNF+ vs. 474.9 ± 563.0 days in the control group; *p* = 0.61).

### 3.3. Feasibility

**Figure 4:**
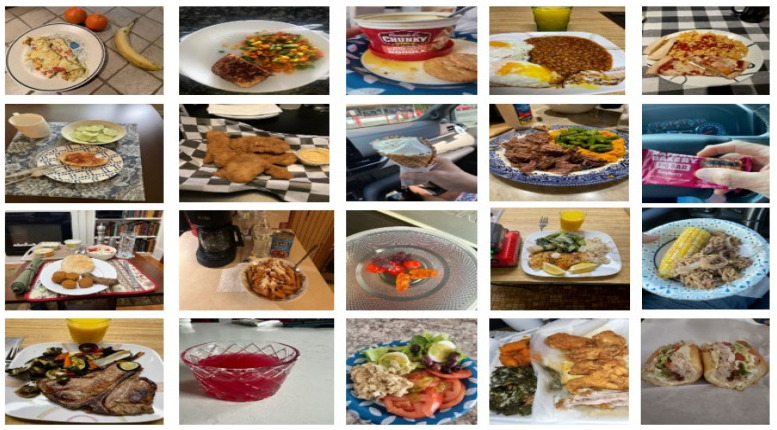
Example food images submitted by PNF+ participants using interactive TXT platform.

Of the 119 eligible participants, 40 were consented and randomized, yielding an accrual rate of 34%. While this did not meet the predefined accrual target of 70%, recruitment challenges in this population were notable and included lack of interest, competing demands, and provider-related factors. Importantly, other key feasibility metrics were achieved. Retention was high, with 83% of participants completing follow-up assessments and a low attrition rate of 5%. Across the PNF+ group 5252 total photos were sent with individual participants sending an average of 228 pictures per their respective intervention period. Examples of food images submitted by the PNF+ group using the interactive TXT platform (Agility Engine Version 4.60.0) are included in Figure 4. Of the 27 participants randomized to PNF+, 24 completed their respective diet regimen. Among the 24 participants, 50% were compliant with 70% of the intervention days with suggested PNF. The health coaching attendance rate for PNF+ was tracked by attendance at scheduled health coaching sessions and classified into categories of low (0–60%), moderate (61–80%), and high (81–100%). Over half of the PNF+ group had high attendance with 58% (*n* = 14), followed by 29% (*n* = 7) in the moderate category and 13% (*n* = 3) in the low category. The average health coaching compliance rate across PNF+ was 82% (*n* = 24) attending all scheduled sessions (Table 2). PNF window compliance was categorized on the same scale as health coaching attendance. In the PNF+ group, the average compliance was 69%, 25% had low adherence with an average of 35% adherence with PNF, 46% fell in the moderate category with an average of 71% adherence with PNF, and 29% had high adherence with an average of 89% adherence with PNF (Table 2). Picture response adherence was categorized on the same scale with 75% of the PNF+ group having high response, 17% having moderate response, and 8% having low response (Table 2).

### 3.4. Acceptability

#### 3.4.1. Severe or Very Severe Adverse Events (A/E)

In terms of acceptability, adverse events were assessed on a biweekly basis in month 1 and weekly in months 2 and 3. Adverse events were only assessed for severity if frequency was above 0, of which were reported. Adverse events were then assessed on a severity scale from 0 to 4, with 0 being none, 3 being severe, and 4 being very severe. Across the intervention period, 554 adverse events were recorded, with 22 recorded as severe or very severe including nausea, fatigue, dizziness, headache, and vomiting. About 4% of recorded adverse events that were assessed for severity were severe or very severe, with fatigue accounting for half of all severe or very severe reported adverse events. These adverse events were determined as unrelated to the study and connected with treatment related side effects, social events, or cold symptoms. Other symptoms were reported during the collection of adverse events, including constipation, urinary incontinence, hot flashes, heartburn, ocular migraine, and cold symptoms. Nearly 12.8% of all other adverse events were severe with cold symptoms, unrelated to the study, accounting for 83.3% of all other reported severe adverse events. Constipation and hot flashes were the most reported adverse events (55.3%) with only one severe case of reported constipation. Constipation was reported by patients as persistent prior to study initiation. Hot flashes were also unrelated to the intervention and reported as a symptom of hormone therapy, which was present prior to study initiation. Adverse events were reviewed by the study team and determined to be unrelated to the intervention. Adverse events were not systematically collected in the control group, which limits between-group comparison.

#### 3.4.2. Client Satisfaction

The average score for client satisfaction did not differ significantly between groups, with the PNF+ group having an average score of 19.8 and the Healthy Diet AC group having an average score of 19.5.

#### 3.4.3. Post-Intervention Interviews

Post-intervention interviews were completed via telephone within at least 2 weeks of intervention completion. Out of the 27 participants randomized for the intervention, 21 completed interviews and were included in the qualitative analysis. Transcripts were analyzed thematically and organized using the COM-B model (Capability, Opportunity, Motivation-Behavior) to identify determinants of adherence to PNF+ and to distinguish barriers from facilitators influencing behavior [21]. See Table 3 for the interview themes organized by the COM-B framework with selected corresponding participants’ quotes.

**Capability domain:** Participants described both psychological and practical factors affecting adherence. A primary barrier was difficulty adjusting to the prescribed fasting window, particularly delaying breakfast or sustaining a full 16 h fast. Some participants reported limited understanding of the intervention purpose at initiation or challenges remembering to log food photographs. In contrast, facilitators included increased awareness of eating patterns, improved understanding of protein intake, and development of self-monitoring skills that enhanced confidence in managing the schedule.

**Opportunity domain:** External, contextual and social influences impact adherence. Work-related demands, business travel, busy daily schedules, and family meal timing were potential challenges. Several participants reported limited control over meal timing due to reliance on spouses for cooking or shared family routines. Social norms, such as no-phone policies at the dining table, also interfered with photo logging. Facilitators included retirement or flexible schedules, staying active outside the home, and enjoyment of cooking, which allowed greater autonomy in structuring meals within the fasting window.

**Motivation domain:** Weight loss, improved energy, metabolic health perceptions, and a sense of control over eating were key facilitators. Many participants valued the accountability provided by the intervention structure and health coaching. They describe coaching as instrumental in reinforcing habits and explaining the rationale behind dietary recommendations. However, few participants reported minimal intrinsic or extrinsic motivation to adhere.

**Behavior:** Participants reported reduced late-night snacking, increased protein intake, improved energy, weight loss, and greater dietary awareness. Most expressed intention to continue PNF in a flexible manner rather than adhering strictly to a 16 h window daily. Several participants described the changes as becoming part of their lifestyle rather than a temporary diet, whereas others planned partial continuation based on personal schedule constraints.

### 3.5. Exploratory Outcomes

#### FACIT-F, FACT-P, Body Weight and BMI

PNF+ participants demonstrated a modest increase in FACIT-F scores over time, with mean scores increasing from 43.6 at baseline to 45.2 at 3 months, indicating reduced fatigue (higher scores reflect less fatigue). The control group showed a similar improvement, with mean scores increasing from 42.5 at baseline to 45.5 at 3 months. For FACT-P, where higher scores indicate better quality of life, participants in the PNF+ group showed an increase from 121.3 at baseline to 125.5 at 3 months, whereas the control group showed a slight decrease from 121.1 at baseline to 119.8 at 3 months. Changes in body weight and BMI were also observed over time. In the PNF+ group, mean body weight decreased from 197.5 to 195.2 lbs, while the control group showed an increase from 220.3 to 223.3 lbs. Similarly, BMI decreased slightly in the PNF+ group (29.3 to 29.1), whereas a small increase was observed in the control group (31.5 to 31.6). Between-group comparisons of change from baseline showed no statistically significant differences across outcomes (all *p* > 0.05). The magnitude of effects was small for body weight and BMI, minimal for FACIT-F, and moderate for FACT-P, although confidence intervals were wide and crossed zero, indicating uncertainty in these estimates. Please see Table 4.

## 4. Discussion

This study found that a 3-month health coaching and interactive TXT-integrated intervention promoting PNF among men receiving ADT demonstrated partial feasibility and high acceptability. With a 34% accrual rate, we did not meet the 70% endpoint goal. This can be attributed to the challenges in recruiting this population on an institutional and study level. However, our study had a considerably low drop-out rate, with a 5% attrition rate. Previous meta-analyses of mHealth interventions found that long-term engagement is a major challenge particularly in interventions that focus solely on chronic disease self-management or diet [22]. While self-management skills may be an important predictor to engagement in mHealth interventions, studies have found that the availability of a health-care professional or health coach increased engagement [22]. Digital interventions can be more convenient and cost-effective, but research shows that face-to-face interaction is something participants are more accustomed to, perceiving interactions as personal, connected, warm and thoughtful [23]. While our study utilized a telehealth format, participants still found the health coach to be a valuable component, cited by some participants as the main driver for consistency. Even with the automated text messages, participants were motivated to know someone was interested in their efforts. This reiterates the importance of personability and connection in digital diet interventions.

Interestingly, the PNF+ group demonstrated an average weight loss of approximately 2.3 lbs from baseline to post-intervention, compared to an average 3.0 lbs gain in the healthy diet group. Although between-group differences were not statistically significant, the direction of change was consistent across several outcomes, with the PNF+ group demonstrating reductions in weight and BMI and improvements in quality of life compared to the control group. Effect size estimates were small for weight and BMI and moderate for FACT-P, suggesting a potential signal of benefit. However, confidence intervals were wide and crossed zero, reflecting uncertainty due to the small sample size. These findings should therefore be interpreted as hypothesis-generating rather than confirmatory. Qualitative findings provide additional context for these patterns, with participants describing increased dietary awareness, accountability through coaching, and improved self-monitoring as key facilitators, while challenges related to fasting schedules, daily routines, and social contexts contributed to variability in adherence. Consistent with these findings, post-intervention interviews highlighted health coaching as a key driver of accountability and motivation. This suggests a potential role for PNF combined with structured coaching support, although findings should be interpreted cautiously given the pilot design. While both groups received automated healthy eating text messages, the additional coaching component in the PNF+ group may have contributed to more favorable weight-related trends. This reinforces the importance of personability and connection in digital interventions. Previous meta-analyses show that personalized and relevant content delivered through text-messaging resulted in higher engagement and an increased sense of ownership over health [23]. Future studies should consider implementing a process, such as a baseline questionnaire or interactive user feedback to inform the tailoring of text-messaging through the intervention in the absence of health coaching.

Several participants plan to continue PNF in some capacity post-intervention, which is an important indicator of feasibility. Participant feedback called for the addition of a follow-up program or extended contact which could serve as a key consideration for larger scale studies to investigate longer-term feasibility and outcomes. Diet interventions inherently require a level of lifestyle change that may not be sustainable outside of the study context when a temporary and external motivator is no longer present. Regains in weight and relapses in health behaviors are common following the end of an intervention, with an average regain of 0.6 lbs per month post intervention [24]. Extending contact with participants following a health coaching intervention through tailored text-messages has been shown to improve regains or relapses, with an average regain of 0.2 lbs per month over a 12-month period compared to the latter [24]. Future studies should consider extending contact with participants through tailored text-messages to assess feasibility of PNF long-term, sustain potential lifestyle change, and better transition participants to the end of a highly interactive study.

Our findings also highlight the influence of spousal dynamics on behavior and lifestyle changes relative to diet quality and meal-timing. Previous research investigating diet quality in a cohort of prostate cancer patients garnered similar results, with the division of food-related responsibilities in the household reflecting traditional gender-roles [25]. Men with healthier diets described their wives as a positive influence on their eating habits, and men with less healthy diets placed partial blame on their wives [25]. Given the important role that wives/partners play in this population’s diet, interventions aiming to change dietary structure or quality among prostate cancer patients should consider including the wives/partners in some capacity.

## 5. Limitations

This pilot study has several limitations that should be considered when interpreting the results. First, the small sample size limits statistical power and precision of effect estimates, as reflected in the wide confidence intervals. As a pilot RCT, the study was not designed to detect definitive between-group differences but rather to assess feasibility and generate preliminary estimates to inform a larger trial. Second, follow-up duration was relatively short, limiting conclusions regarding long-term sustainability of dietary adherence and clinical outcomes. Third, outcome measures such as weight were collected during routine clinical visits, which may have introduced variability in timing across participants. Future adequately powered trials should incorporate longer follow-up and include additional objective clinical outcomes, such as, metabolic biomarkers, body composition measures, inflammatory markers, and disease-specific endpoints to better evaluate the physiological and oncologic impact of prolonged nightly fasting. Additionally, because the intervention combined prolonged nightly fasting with structured telehealth coaching, the independent contribution of each component cannot be determined. A baseline imbalance in household income was observed and may reflect underlying socioeconomic differences that could influence behavioral outcomes. The study was not powered to detect differences in exploratory outcomes, and variability in measures such as medication duration may have contributed to heterogeneity in results.

## 6. Conclusions

In conclusion, PNF+ demonstrated partial feasibility and high acceptability among men receiving ADT for prostate cancer. Preliminary findings suggest potential benefits in body weight, with more modest changes observed in BMI, and favorable trends in fatigue and quality of life. While between-group differences were not statistically significant, the overall direction of effects supports further investigation. Given the pilot nature of this study and the small sample size, these findings should be interpreted as hypothesis-generating. A larger, adequately powered randomized controlled trial with longer follow-up is warranted to confirm efficacy, further evaluate clinical outcomes, and refine intervention components, including behavioral support, personalized messaging, and consideration of household and social dynamics.

## Figures and Tables

**Table 1:** Baseline demographic and clinical characteristics.

Demographic Variable	All (*n* = 40)	PNF+ (*n* = 27)	Healthy Diet (*n* = 13)	*p*-Value
**Mean Age & SD**	69.5 ± 7.6	69.0 ± 6.3	70.3 ± 10.0	0.68
**Race**	%	%	%	0.86
*Black or African American*	32.5%	33.3%	30.8%	
*White*	62.5%	63.0%	61.5%	
*Other*	5.0%	3.7%	7.7%	
**Education**	%	%	%	1.00
*College Graduate or Post-College*	47.5%	48.1%	46.2%	
*Below College*	52.5%	51.9%	53.8%	
**Household Income**	%	%	%	0.02
$0–$15,000	12.5%	14.8%	7.7%	
$15,001–$60,000	40.0%	40.7%	38.5%	
*$60,001*–*$75,001*	10.0%	0.0%	30.8%	
*$75,001+*	37.5%	44.4%	23.1%	
**Mean Medication Duration in Days**	403.9 ± 590.0	368.0 ± 604.8	474.9 ± 563.0	0.61
**Hormone Therapy Medication**	%	%	%	N/A
LHRH Agonist	82.5%	85.2%	76.9%	
LHRH Antagonist	10.0%	11.1%	7.7%	
Androgen Receptor Inhibitor	27.5%	29.6%	23.1%	
Androgen Synthesis Inhibitor	15.0%	11.1%	23.1%	
Bone Modifying Agent	5.0%	7.4%	0.0%	

Continuous variables are presented as mean ± standard deviation. Categorical variables are presented as percentages. Continuous variables were compared using independent *t*-tests and categorical variables using Fisher’s exact tests. Given the small sample size, *p*-values are presented for descriptive purposes only. Categories for medication use are not mutually exclusive; therefore, percentages may exceed 100%.

**Table 2:** Adherence to health coaching, PNF window, and picture response in the PNF+ group.

	Health Coaching Adherence		PNF Window Adherence		Picture Response Adherence	
	% of Sample	Mean (%)	% of Sample	Mean (%)	% of Sample	Mean (%)
**Low (0–60%)**	13%	42%	25%	35%	8%	55%
**Moderate (61–80%)**	29%	64%	46%	71%	17%	73%
**High (81–100%)**	58%	89%	29%	89%	75%	92%
**Total**	100%	82%	100%	69%	100%	85%

**Table 3:** Post-intervention interview themes organized by COM-B framework.

Domain	Category	Barriers/Facilitator	Sample Participants Quotes
Capability	Psychological capability	Barrier	*“I just couldn’t adjust to the fasting period… I couldn’t wait until 9 o’clock to eat breakfast.” “Was not fully aware of the purpose at the beginning.”*
		Facilitator	*“It helps me make more conscious decisions and be aware of how I feel when I eat.” “Will eat more protein for breakfast, I am more conscious of the diet.”*
	Practical capability	Barriers	*“I forgot to send pictures most of the times.”*
Opportunity	Practical opportunity	Barrier	*“It is difficult to do, especially with business travel.” “I don’t finish work until 6:30 p.m.” “If you are on a rigid work schedule, I think it would work.”*
		Facilitator	*“I am retired so the schedule suits me.” “Being out of the house and being active helps.” “I enjoy cooking… I like the schedule.”*
	Social opportunity	Barrier	*“If I radically alter mealtimes, it would have caused disruption.” “I do not do the cooking; my wife does.” “We have this rule that no phones are allowed at the dining table.”*
Motivation	Perceived benefits	Facilitator	*“The results are very appealing; weight loss and energy are appealing.” “It gave my body some time to rest.” “It makes you aware of your eating habits and keep you accountable*
	Habit and emotional reinforcement	Facilitator	*“Cutting out snacks at night made me feel so much better.” “I don’t sit down with a bowl of ice cream anymore.”*
	Intervention support (coaching)	Facilitator	*“The coaching was helpful… it helps form habits.” “I wouldn’t have been as consistent without talking to someone.”*
	Low motivation	Barrier	*“I was not extrinsically motivated, and I was not intrinsically motivated.”*
Behavior	Behavioral outcome	Facilitator	*“I am going to continue doing this forever.” “Plan to continue but probably not so strict with it.” “Again, I may not make the full 16 h every day.” “This program made me change.”*

**Table 4:** Changes in clinical and patient-reported outcomes from baseline to 3 months.

Outcome	PNF+ (*n* = 23)		Healthy Diet (*n* = 10)		Mean Difference in Change from Baseline	95% CI for Difference	*p*-Value	Effect Size (Cohen’s d)
	Baseline Mean (SD)	Post-Intervention Mean (SD)	Baseline Mean (SD)	Post-Intervention Mean (SD)				
**Body Weight (lbs.)**	197.5 (24.2)	195.2 (25.7)	220.3 (28.1)	223.3 (29.5)	−2.7	(−9.3, 3.9)	0.41	0.31
**Body Mass Index (BMI)**	29.3 (2.9)	29.1 (3.0)	31.5 (5.6)	31.6 (5.8)	−0.2	(−0.9, 0.5)	0.32	0.38
**FACIT-F**	43.6 (5.5)	45.2 (5.3)	42.5 (10.1)	45.5 (11.2)	−1.4	(−4.6, 1.8)	0.56	−0.25
**FACT-P**	121.3 (14.0)	125.5 (12.0)	121.1 (21.3)	119.8 (22.6)	5.5	(−3.0, 14.0)	0.10	0.72

Continuous variables are presented as mean (SD) at baseline and 3 months. Mean difference represents the between-group difference in change from baseline based on individual change scores. *p*-values were calculated using independent *t*-tests comparing change scores between groups. Ninety-five percent confidence intervals (95% CI) are reported for between-group differences. Effect sizes are presented as Cohen’s d based on change scores. Sample sizes vary across outcomes due to missing data; analyses were conducted using available cases, excluding invalid values.

## Data Availability

The original contributions presented in this study are included in the article Further inquiries can be directed to the corresponding author.

## Abbreviations

The following abbreviations are used in this manuscript:

PCa	Prostate Cancer
ADT	Androgen Deprivation Therapy
QOL	Quality of Life
PCSM	Prostate Cancer Specific Mortality
DALYS	Disability-adjusted Life Years
PNF	Prolonged Nightly Fasting
RCT	Randomized Controlled Trial
IGF-1	Insulin-like Growth Factor
CVD	Cardiovascular Disease
EMR	Electronic Medical Records
GNRH	Gonadotropin Releasing Hormone
BMI	Body Mass Index
CSQ-8	Client Satisfaction Questionnaire-8 Item
A/E	Adverse Events
FACIT-F	Functional Assessment of Chronic Illness Therapy-Fatigue
FACT-P	Functional Assessment of Cancer Therapy-Prostate Cancer
